# Advanced Sensing Technology for Ocean Observation

**DOI:** 10.3390/s25041228

**Published:** 2025-02-18

**Authors:** Federico Angelini

**Affiliations:** Italian National Agency for New Technologies, Energy and Sustainable Economic Development (ENEA), Laboratory FSN-TECFIS-DIM, 00044 Frascati, Italy; federico.angelini@enea.it

## 1. Introduction

It is almost impossible to overestimate the importance of the oceans for human society and the whole biosphere, either from the perspectives of climate change or sustainable development [1]. On the one hand, reciprocal interactions between the atmosphere and the oceans are recognized to have a key role in the climate change and to act through very complex feedbacks [2,3] and tipping points [4]. On the other hand, sustainable development is tightly connected to a conscious exploitation of the oceans [5,6,7]; just think of the impact of pollution, transport, and fisheries on human health and the whole society.

It is widely agreed among the scientific community, as well as among policymakers, that a deep understanding of the ocean ecosystem is required to create figure out and implement mitigation and adaptation plans [8].

It has long been known that the oceans play a key role in determining climate conditions and life on the Earth [9]. For many decades, scientists have been raising the alarm on the health of the oceans [10] and their impact on climate change [11]. Marine ecosystems are changing quickly, at unprecedented rates [12] in terms of physical, chemical, and biological characteristics [13]. Nevertheless, it is not easy to detect and quantify all these changes [14].

In fact, despite its importance, many aspects of sea science are still relatively unknown [15], both because of the complexity of interactions between physics, chemistry, biology, and human activities, and because of the difficulties inherent in carrying out accurate, continuous, and reliable measurements of even simple variables such as temperature and salinity. Many kinds of remote and/or automated measurements, conducted using technology such as satellites, offer great advantages in terms of coverage and continuity, but suffer important drawbacks that cannot be overcome through other satellite observations (for example, vertical profiles in water are inaccessible using remote sensing because of the strong absorption of electromagnetic waves by water).

Many current problems, spanning different spatial scales (from molecular processes to global issues) and timescales (from chemical dynamics to geological eras), still limit our understanding of the processes that involve ocean physics, chemistry, and biology. Accurate, reliable, and continuous measurements in a variety of fields [16,17,18], from basic physics to applications for the optimization and sustainability of fishing and navigation, present new challenges. This Special Issue aimed to gather valuable and innovative papers on a wide range of new methods and technologies to improve the quality and resolution of oceanographic data, and to integrate different data sources.

## 2. Overview of Published Papers

This Special Issue gathers together nine research papers and one technical note, discussing new progresses in marine sensing in a wide range of technologies. These are summarized below, sorted by publication date.

In Contribution 1, Zhang et al. present a new method for creating mosaics from striped images obtained by side-scan sonar technology. The problem addressed is the distortion and different resolution of overlapping areas in images, typical of complex marine environments. The proposed solution is based on curvelet transform, a multi-scale analysis method more effective than the wavelet transform for the representation of details and edges in images. First, a register of the images is made to eliminate distortions and dislocations, and then a resolution vector for overlapping areas is calculated and a resolution weight model is used to guide the fusion of the curvelet coefficients. Eventually, the inverse curvelet transform generates the final mosaic. Experiments with real data demonstrate the superiority of the proposed method over traditional image fusion algorithms.

In Contribution 2, Jutard et al. describe a new quality control protocol (“delayed-mode quality control”) for radiometry data acquired by the Biogeochemical-Argo (BGC-Argo) floats. The main problem addressed is the correction of systematic errors in data due to the temperature dependence and time drift of irradiance sensors. The authors propose a method to correct these errors using auxiliary night measurements and daily measurements at 1000 dbar, validating the method on over 10,000 profiles from various ocean regions. The aim is to improve the quality of BGC-Argo’s radiometry data, making them more accurate and reliable for oceanographic research and, in particular, for studying phytoplankton dynamics and integrating with satellite observations.

In Contribution 3, Wassim Baba et al. present, as a technical note, an innovative methodology for obtaining large-scale coastal bathymetry using Sentinel-2 satellite imagery and a high-performance cluster (HPC). The main objective is to overcome the limitations of traditional, time-consuming, and costly techniques for mapping coastal waters. The researchers describe an approach that uses the properties of ocean waves, visible in Sentinel-2 images, to estimate water depth, implementing it on an HPC to handle the high volume of data. The case study focuses on the North African coast, comparing results obtained with a reference bathymetric product (GEBCO), highlighting the potential of the method to create high-resolution global bathymetric maps. The methodology therefore offers a more efficient and cost-effective approach to monitoring coastal areas, which are crucial for the environment and economic development.

In Contribution 4, Li et al. describe an innovative method for estimating water transport in an Arctic lagoon connected to the Beaufort Sea. Using a combination of short-term measurements from a ship-mounted acoustic Doppler current profiler (ADCP) and long-term measurements from a bottom-anchored ADCP, the researchers established a statistical relationship between the measured water velocity and total transport. This approach, validated with a coefficient of determination R2 of 0.89, allows the estimation of water transport over longer periods, overcoming the constraints imposed by the harsh environmental conditions of the Arctic and limited resources. The study highlights the importance of measuring water transport in the Arctic regions to understand the impact of climate change and provides an effective and cost-effective methodology for addressing this challenge.

In Contribution 5, Wang et al. present an evaluation of a new lightweight mouse-bathymetric LiDAR system mounted on a drone (UAV), called Mapper4000U. The study compares the bathymetric performance of the Mapper4000U with that of a LiDAR system mounted on a manned aircraft, using data from a Chinese coastal island and data from a multibeam sonar as a reference. The main objective is to demonstrate the Mapper4000U’s ability to perform high-resolution bathymetric mapping in shallow water, including underwater object detection. The results show a high precision and accuracy of the UAV system, with a significantly higher point density than the traditional LiDAR system, while maintaining a good penetration depth. The article examines in detail the data processing methodology and discusses the environmental effects on measurements.

In Contribution 6, Nekrasov et al. present research on the optimization of sea wind measurements using an aerial scatterometer with a rotating antenna mounted under the fuselage. The study focuses on the analysis of normalized radar cross-section sampling at different incidence angles, assessing the accuracy of wind vector estimation using a single angle or combinations of angles. The results of the Monte Carlo simulations show that using more than one close angle of incidence significantly improves measurement accuracy while reducing maximum operating altitude. The work aims to improve the functionality of existing airborne radar and to drive the development of new remote sensing systems for measuring sea wind.

In Contribution 7, Nagano et al. describe an experiment conducted off the coast of Sanriku, Japan to study the turbulent heat flux between the ocean and the atmosphere. Using an autonomous marine vehicle, a Wave Glider, the researchers measured various parameters (air and sea temperature, humidity, wind speed) for 55 days to calculate heat flow, focusing in particular on sub-mesoscale variations. The study shows that the intrusion of cold and dry air masses, following the passage of low atmospheric pressure systems, generates a significant heat flux upwards over relatively warmer water regions, highlighting the importance of high-resolution observations to fully understand the ocean–atmosphere interaction and improve weather and climate forecasts. The results show that satellite observations, due to their low resolution, may underestimate the influence of sub-mesoscale variations in heat flux.

In Contribution 8, Hoffman et al. present a new, simple, and economical method for estimating the coefficient of resistance of submerged floats, in particular focusing on those with complex and non-rigid shapes, which are difficult to model using analytical or numerical methods. The authors propose an in-situ approach based on the relationship between the float’s attitude speed and its weight, varied by adding ballast. The accuracy of the method in obtaining the coefficient of resistance, hydrostatic strength, and, if present, the force of the thruster is demonstrated by experiments at sea. The simplicity of the method makes it applicable to arbitrarily shaped objects, overcoming the limitations and costs of conventional methods such as CFD simulations or dry-dock tests.

In Contribution 9, Nie et al. describe the development and use of a long-range hybrid Autonomous Underwater Vehicle (AUV) to measure ocean turbulence. The unique AUV combines the features of a traditional AUV and a variable-floating glider, offering different flexible movement modes. It was deployed for continuous measurements in the northern part of the South China Sea, collecting high spatial and temporal resolution data on turbulence and its relationship with thermocline, highlighting how the latter acts as a “barrier” against the transmission of energy from the surface level to greater depths. The study shows the usefulness of these hybrid AUVs as a powerful tool for studying ocean turbulence on a large scale.

In Contribution 10, Sattar present a new autonomous acoustic method for the identification of vocalizations of endangered whales, focusing on blue whales and common whales. The proposed method combines wavelet scattering transform with a deep-learning LSTM classifier, demonstrating high classification accuracy (over 97%) even with limited data sets. This represents a significant improvement over existing methods, making it possible to monitor the acoustic performance of these species more efficiently for conservation purposes, allowing better tracking of their numbers, migratory routes, and habitats. The research highlights the importance of artificial intelligence and deep learning in marine acoustic data analysis for whale protection.

## 3. Conclusions

The published papers cover a range of applications of signal processing and machine learning techniques for the analysis of data from marine environments. The works also highlight the importance of developing new methods and technologies to improve the quality and resolution of oceanographic data, as well as integrating different data sources for a more complete understanding of ocean processes.

The wide range of topics discussed in this Special Issue bears witness to the importance of improvements in ocean monitoring and the need for a better understanding of all the processes involving biology, chemistry, and physics in the oceans to better understand, in turn, connections with climate change and to plan the best strategies for mitigation and adaptation.
